# Robust Latent Multi-Source Adaptation for Encephalogram-Based Emotion Recognition

**DOI:** 10.3389/fnins.2022.850906

**Published:** 2022-04-27

**Authors:** Jianwen Tao, Yufang Dan, Di Zhou, Songsong He

**Affiliations:** ^1^Institute of Artificial Intelligence Application, Ningbo Polytechnic, Ningbo, China; ^2^Industrial Technological Institute of Intelligent Manufacturing, Sichuan University of Arts and Science, Dazhou, China

**Keywords:** encephalogram, latent space, emotion recognition, co-adaptation, maximum mean discrepancy

## Abstract

In practical encephalogram (EEG)-based machine learning, different subjects can be represented by many different EEG patterns, which would, in some extent, degrade the performance of extant subject-independent classifiers obtained from cross-subjects datasets. To this end, in this paper, we present a robust Latent Multi-source Adaptation (LMA) framework for cross-subject/dataset emotion recognition with EEG signals by uncovering multiple domain-invariant latent subspaces. Specifically, by jointly aligning the statistical and semantic distribution discrepancies between each source and target pair, multiple domain-invariant classifiers can be trained collaboratively in a unified framework. This framework can fully utilize the correlated knowledge among multiple sources with a novel low-rank regularization term. Comprehensive experiments on DEAP and SEED datasets demonstrate the superior or comparable performance of LMA with the state of the art in the EEG-based emotion recognition.

## Introduction

Contemporarily, in the field of affective computing research, automated emotion recognition (AER) has attracted lots of attention from machine learning and computer vision (Kim et al., 2013). In traditional schema, one auto emotion recognition system driven by EEG signals usually includes two core components, i.e., feature extraction followed by emotion classification (Lan et al., 2018). Some representative EEG feature extraction methods (Jenke et al., 2014; Zhang et al., 2020b) can be viewed comprehensively in Jenke et al. (2014). This work mainly focuses on machine learning-based emotion classification methods.

In the past decade, a large scale of emotion recognition methods has been presented for effective emotion recognition using EEG features (Musha et al., 1997; Kim et al., 2013; Li et al., 2018b,c). Zheng (2017) proposed a novel emotion recognition method by exploiting the group sparse canonical correlation analysis, thus simultaneously implementing EEG channel selection and emotion recognition. Recently, Li et al. (2018c) also presented a sparse linear regression model with graph regularization for emotion recognition using EEG signals. In the past decade, due to their outperformed performance compared with traditional methods, deep emotion recognition methods using EEG signals have been widely explored in emotion feature extraction and recognition (Lotfi and Akbarzadeh-T, 2014), such as criminal psychological emotion recognition based on deep learning and EEG signals (Liu and Liu, 2021), EEG-based Deep Belief Network model (Zheng and Lu, 2015), multi-channel EEG-based recognition model (Song et al., 2018), and EEG-based neural network model (Li et al., 2018b).

It is worthy to note that the aforementioned works for emotion recognition perform well only in such scenario that both training and test samples follow the same distribution (Zhang et al., 2020a), in which the recognition models obtained from the source dataset(s) therefore can be easily utilized in the target dataset effectively (Zhang et al., 2019a). Unfortunately, these traditional methods may fail in addressing cross-subject/dataset emotion recognition due to the mismatch of feature distribution with EEG signals. To address this issue, many domain adaptation (DA) emotion recognition models for AER problem have been promoted (Chu et al., 2017; Li et al., 2018a; Li et al., 2020a,b; Bao et al., 2021; Wang et al., 2021). In a DA emotion recognition system, one usually focuses on exploring an effective recognition model on one target domain with few or even none of the labeled data, by borrowing some positive knowledge from other source domain(s) with slightly different distribution with that of the target domain (Bruzzone and Marconcini, 2010; Tao et al., 2012; Long et al., 2014; Zhang et al., 2019b).

A typical challenge in one EEG-based emotion recognition system is the cross-subject/dataset learning problem (Li et al., 2018a). In such scenario, DA techniques can be exploited to address this challenging issue where both training and test data follow slightly different distribution (Tao et al., 2012; Long et al., 2014; Li et al., 2021). To deal with the challenging cross-subject EEG emotion recognition problem, Pandey and Seeja (2019) proposed a subject-independent approach for EEG emotion recognition. Li et al. (2018a) proposed another method for cross-subject EEG emotion recognition. In the past decade, deep neural networks (DNNs) (Ganin et al., 2016; Li et al., 2018a) have also driven rapid progress in DA (Duan et al., 2012a; Ding et al., 2018a). The DA issues can be solved by the domain adversarial neural network (DANN) (Ganin et al., 2016). It remains unclear, however, whether the performance of deep DA methods is really contributed by their deep feature representation, the fine-tuned classifiers, or is rather an outcome of the adaptation regularization terms (Ghifary et al., 2017).

Although existing DA methods have obvious effectiveness and efficiency in the special use of emotion recognition (Chu et al., 2017), there is few work to use the joint feature selection method and then carry out the multi-source adaptive domain recognition of cross datasets by exploiting the correlation knowledge among domains and features. Besides, during DA, most of the multi-source domain adaptation (MDA) methods (Yang et al., 2007; Duan et al., 2012b,c; Tommasi et al., 2014; Tao et al., 2015, 2017; Ding et al., 2018b) generally cope with the sources independently without considering the correlation information among the source domains (Zhang et al., 2019c), which may destroy the discriminant structure (either intrinsic or extrinsic) of multi-source domains (Rosenstein et al., 2005). Last but not the least, for an MDA system, it is crucial for source weight determination during learning based on the correlation and quality of source domains. To the best of our knowledge, these characters are not feasible enough in extant MDA methods.

In order to solve the above problems in existing MDA, we explore to exploit the relevant knowledge among sources in the uncovered subspaces to learn a multi-source adaptive emotion recognition model. In other words, we mainly adopt the strategy of digging the relationship between multi-source domains and one target domain (including feature and distribution) for promoting multi-source adaptive emotion recognition with EEG signals. We aim to progress beyond existing works that have partially addressed those issues by exploring to solve all the above-mentioned issues in a unified framework. Specifically, we propose in this work a robust Latent Multiple-source Adaption (LMA) method for EEG-based emotion recognition by mining multiple shared latent subspaces, each for one source–target domain pair. The method employs the robust regression scheme to process high-dimensional, sparse outliers and non-i.i.d. (independently identical distribution) EEG features by jointly utilizing the *l*_2,1_-norm (Nie et al., 2010a) and trace norm. Under this framework, the row sparsity regularization is designed to obtain the solution of sparse feature selection (Zhang et al., 2020b). We match distributions between each domain pair (including both target and multi-source domains) by minimizing the nonparametric Maximum Mean Discrepancy (MMD) (Gretton et al., 2009; Pan et al., 2011) in each uncovered latent space shared by this source–target pair. The contributions of this paper are listed as follows:

(1) We propose a unified multi-source adaptive emotion recognition framework with EEG features by uncovering multiple latent subspaces.
(2) Our framework selects features in a collaborative way and considers the correlated knowledge among sources. In LMA, the importance of each feature does not need to be evaluated separately. In addition, in our unified framework, we can learn multiple loss functions with feature selection for all source adaptation subjects synchronously, so that our framework can use the correlated information of multiple sources as auxiliary information.
(3) In this framework, the original geometric structure is retained by using the graph Laplacian regularization, and the *l*_2,1_-norm minimization sparse regression approach is used to suppress the influence of noise or outliers in the domains, which shows the robustness of the framework.
(4) Through a large number of experiments on two EEG datasets, we prove the effectiveness and convergence of this framework.

The remainder of the paper is organized as follows: In section Related Work, we discussed the related works with feature selection and multi-source DA learning. In section Proposed Framework, our framework LMA will be designed, and section Algorithm arranges the corresponding optimal algorithm of LMA. The experimental results and analysis on two real EEG datasets are presented in section Experimental Evaluation. Finally, we conclude in Section Conclusion.

## Related Work

In the past decades, affective computing community has paid increasing attention to the emotion recognition with brain–computer interfaces (BCI) (Mühl et al., 2014; Chu et al., 2017). A brain–computer interface system could capture certain emotion states and respectively make corresponding response to these states using spontaneous EEG signals even when explicit input from the subjects is unavailable (Zhang et al., 2019a), thus augmenting the user experience in the session of interactivity. Nowadays, a large number of methods (Zhang et al., 2016, 2017) have been proposed to recognize different emotion information from brain-wave signals. The latest works about affective BCI (aBCI) took account of machine learning algorithms on emotion recognition using a few discriminative features (Jenke et al., 2014; Mühl et al., 2014). In one representative BCI system, a certain feature extractor firstly extracts discriminative features from the raw EEG data, and then these features as well as labeled emotion states are sent into the classifier for real-time affection recognition. In the last decade, many aBCI-related works have presented sound and interesting emotion recognition performance (Mühl et al., 2014).

Although existing methods have obtained satisfied achievements on EEG-based emotion recognition, the expected performance could still be degraded by certain impacts in the case of cross-subject/dataset recognition due to the difference between subjects/datasets. Therefore, one needs to train a specific classifier for individual subject/dataset-of-interest. Even for the same subject, it is also indispensable to recalibrate the classifier frequently for maintaining a satisfied recognition accuracy since the EEG signals are unstable now and then. This would undoubtedly increase the costs of manual labor as well as time. Fortunately, the DA (a.k.a. domain transfer) technique can be leveraged to tackle these issues existing in EEG-based emotion recognition.

In the past decade, DA technique (Duan et al., 2012a,b,c; Tzeng et al., 2015; Tzeng et al., 2017; Ding et al., 2018a,b,c) has elicited an increasing attention in the community of machine learning. Up to now, domain-adaptation-based emotion recognition methods have nearly dominated the literature of aBCI Dolan, 2002; Mühl et al., 2014; Jayaram et al., 2016; Lan et al., 2018; Zhong et al., 2020; Zheng et al., 2015; Zheng and Lu, 2016; Chai et al., 2016; Chai et al., 2017; Shi et al., 2013; Koelstra et al., 2012; Zheng and Lu, 2015), which aim to address different issues in emotion classification by pursuing various DA skills using the EEG datasets such as SEED. In these preceding works, a commonly used strategy is to uncover a shared subspace from different domains by preserving certain discriminative properties, thus decreasing the differences among subjects or sessions extracted from the captured EEG signals (Tao and Dan, 2021). While extensive exploration on cross-subject/session has been conducted effectively in the prior works by leveraging various DA tricks, one obvious shortage in these works is that the evaluation dataset is just limited to one single database, e.g., SEED. In practical aBCI applications, the EEG datasets could also change since the EEG signals may be produced by different subjects, sessions, EEG devices, experimental schemes, and emotional stimuli. Henceforth, one of the yet unsolved issues in current research is the robustness and effectiveness of the proposed DA methods on cross-datasets/subjects.

## Proposed Framework

### Notation

In the context, the symbol definitions are listed in Table 1. We respectively denote by [*A*_1_, *A*_2_, …, *A*_*k*_] and [*A*_1_; *A*_2_; …; *A*_*k*_] the concatenation of *k* matrices according to the row (horizontally) and the column (vertically). In this work, we focus on the multi-source adaptation framework, which can be driven by *S* source domains of *c*-class. We denote by $X^{a}=\left\{x_{1}^{a},\ldots ,x_{n_{a}}^{a}\right\}\in {\mathbb{R}}^{d\times n_{a}}$ (*a* = 1, 2, …, *S*) the *a*th source dataset with *n*_*a*_ samples^1^. Its corresponding class label matrix can be denoted as $Y^{a}={\left[y_{1}^{a},\ldots ,y_{n_{a}}^{a}\right]}^{T}\in {\mathbb{R}}^{n_{a}\times c}\in \Gamma ={\left\{0,1\right\}}^{c\times 1}$ with *y*_*il*_ = 1 if the *i*th sample is labeled as the *l*th class and -1 others. Correspondingly, we denote by $X^{t}=\left\{x_{1}^{t},x_{2}^{t},\ldots ,x_{m}^{t}\right\}\in {\mathbb{R}}^{d\times n_{t}}$ the target dataset of interest. Since the true classes of the samples in *X^t^* are inaccessible in the training stage, the target labels (or pseudo labels) $Y^{t}={\left[y_{1}^{t},\ldots ,y_{n_{t}}^{t}\right]}^{T}\in {\mathbb{R}}^{n_{t}\times c}\in \Gamma$ can be predicted by certain pre-trained classifiers trained on the source datasets with labeled data. Therefore, detecting the ground-truth label of each target sample is our ultimate goal.

**TABLE 1:** Notations and descriptions.

Notations	Descriptions
*N*	Sample number of each source–target pair
*d*	Feature dimensionality number
χ	Sample/feature space
Γ	Label/prediction space
*a* = [*a*_1_, *a*_2_, …, *a*_*d*_]^*T*^ ∈ ℝ^*d*^	Vector *a*
*A* ∈ ℝ^*n*×*d*^	Matrix *A*
*A*_*i*, *j*_	The (*i*, *j*)th element of the matrix *A*
*A*_*i*,:_ and *A*_:, *j*_	The *i*/*j*th row/column vector of *A*
(⋅)^*T*^	Transpose operator
*tr*(⋅)	Trace operator
⟨*A*, *B*⟩ = *tr*(*A^T^B*)	The inner product of two matrices *A* and *B*
${||a||}_{p}:={\left(\sum_{i=1}^{d}{\left|a_{i}\right|}^{p}\right)}^{1/p}$	The *p*-norm of a vector *a*
${||A||}_{2,1}=\sum_{i=1}^{n}{||A_{i,:}||}_{2}=\sum_{i=1}^{n}\sqrt{\sum_{j=1}^{d}A_{ij}^{2}}$	The *l*_2,1_-norm of *A*
${||A||}_{*}=tr\left({\left(AA^{T}\right)}^{\frac{1}{2}}\right)$	The trace-norm of *A*
*I*_*r*_	Identity matrix of size *r*×*r*
1_*d*_	*d*-dimensional vector with all ones
0_*d*_	*d*-dimensional vector with all zeroes

We further denote *X^a^*/*X^t^* with the label $\bar{l}$ as $X^{a\left(\bar{l}\right)}$/$X^{t\left(\bar{l}\right)}$ ($\bar{l}=1,\ldots ,c$), and the *a*th source–target domain pair as *X*_*a*_ = [*X^a^*, *X^t^*] ∈ ℝ^*d*×*N*^(*N* = *n*_*t*_ + *n*_*a*_) with label matrix *Y*_*a*_ = [*Y^a^*, *Y^t^*] by packing the *a*th source and the target data.

### Problem Statement

A commonly used strategy in the representative MDA is to acquire knowledge from multiple sources by leveraging certain common knowledge shared by them to promote the target learning of interest. We propose in this work a robust Latent Multiple-source Adaption (LMA) emotion recognition method based on EEG features. The method employs the robust regression scheme to process high-dimensional, sparse, outliers, and non-i.i.d. EEG features by jointly utilizing the *l*_2,1_-norm and trace norm (Yang et al., 2013). The designed method has three characteristics, which are integrated into a unified optimization formulation to find an effective emotion recognition model by aligning the feature distribution between each source–target domain pair. Specifically, it includes four technical aspects: (1) *via* employing the *l*_2,1_-norm minimization, a robust loss term is introduced into each source model learning by taking account of the influence of noise or outliers in EEG signal (Li et al., 2015), and a sparse regularization term is designed to eliminate over-fitting and a sparse features subset is selected; (2) based on the designed regression model and the semantic distribution matching between each pair of domains in each uncovered latent spaces (Tao et al., 2019), it not only provides robustness on loss function, but also retains the domain distribution (including local and global) structures (Nie et al., 2010b), and meanwhile maintains a high dependence on the (pseudo) label knowledge of the source domains and the target domain (Nie et al., 2010b; Ding et al., 2018c; Zhang et al., 2020a), so as to obtain preferable generalization performance; and (3) by exploiting the trace norm of matrix, we can make full use of the correlative information among multiple sources and transfer more discriminative knowledge to the target domain.

**FIGURE 1:**
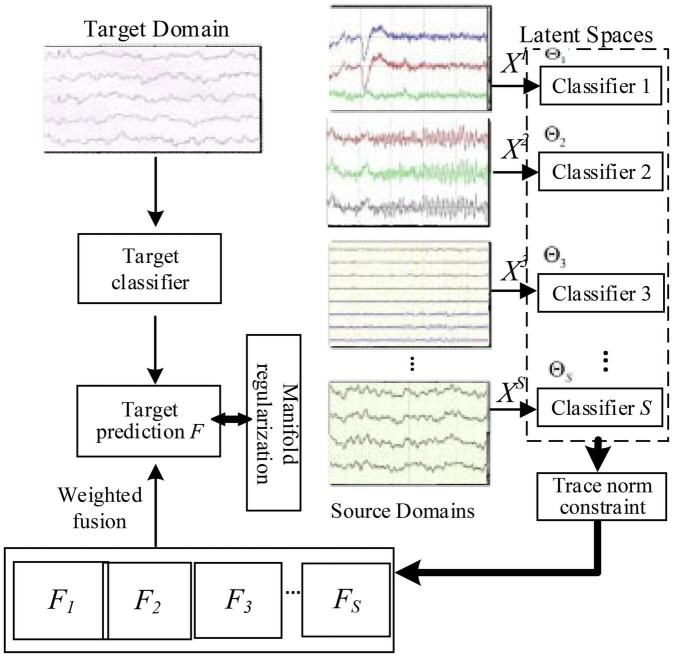
Flowchart of LMA on EEG-based emotion recognition.

Specifically, we present the flow diagram of LWA in Figure 1 to illustrate our innovation: firstly, we can project each source EEG data into one domain-invariant subspace by minimizing the domain-wise distribution discrepancy; thus, *S* classifiers are being jointly learned by employing trace norm as well as *l*_2,1_-norm; we then obtain *S* target label matrices predicted from these source classifiers on the target domain; furthermore, in the original space, we also learn a target model using the squared regression scheme with the constraint of prediction consistency on the target data between those source models and the target model; and by uncovering multiple domain-invariant latent spaces, we finally formulate a joint learning framework of multi-source adaptation for EEG-based emotion recognition. To implement these properties, in the following part, we will detail the objective formulation of the proposed method.

### General Formulation

In this section, we propose the general formulation of LMA framework underpinned by the robust regression principle and the regularization theory. We investigate the learning problem under multiclass setting, with the decision classifiers ${\left\{f_{\theta }^{a}\left(x\right)\right\}}_{a=1}^{S}$, where θ is the parameter of the hypothesis space of those decision functions. We propose a unified MDA framework by uncovering *S* discriminative latent subspaces Θ_*a*_ (*a* = 1, 2, …, *S*) (Tao and Xu, 2019) and to learn decision classifiers $f_{\theta }^{a}\left(x\right)$s of all sources simultaneously. In particular, the proposed method minimizes the distribution difference of each domain pair after the projection *P*_*a*_ into the subspace Θ_*a*_, as well as the structural risk functional of the labeled data from the source domain *X^a^*. We also let Θ_*a*_ be orthogonal on rows so that $\Theta_{a}\Theta_{a}^{T}=I_{r\times r}$, where *r*(≪*d*) is the dimensionality of the shared latent space. We then endeavor to find *S* cross-domain models parameterized by ${\left\{\theta_{a}\right\}}_{a=1}^{S}$ *via* jointly utilizing correlated knowledge among sources in some latent spaces. We therefore propose the following general formulation of LMA:


(1) $$\begin{array}{c} \begin{array}{c} ℜ\left(f_{\theta_{a}}^{a},f_{\theta_{t}}^{t},\Theta_{a}\right)\\ =\sum_{a=1}^{S}\left(R\left(f_{\theta_{a}}^{a},Y_{a}\right)+dist_{\Theta_{a}}\left(X^{a},X^{t}\right)+℧\left(f_{\theta_{a}}^{a}\right)\right)+Con\left(f_{\theta_{a}}^{a},f_{\theta_{t}}^{t},X^{t}\right) \end{array}\\ \;\begin{array}{c} +R\left(f_{\theta_{t}}^{t},Y^{t}\right)+\Omega \left(f_{\theta }^{a}\right) \end{array}, \end{array}$$

where θ_*a*/*t*_ is the parameter set of the *a*th source/target model, $Con\left(f_{\theta_{a}}^{a},f_{\theta_{t}}^{t},X^{t}\right)$ enforces the discriminative consistency between the *a*th source and the target model on the target dataset, *R*(⋅,⋅) is the robust regression model, the regularization term $℧\left(f_{\theta_{a}}^{a}\right)$ controls the complexity of $f_{\theta_{a}}^{a}$, *dist*_Θ_*a*__(*X^a^*, *X^t^*) is for aligning the distributions of each domain pair in the latent space Θ_*a*_, the regularizer $\Omega \left(f_{\theta }^{a}\right)$ controls the low rankness of all source models for mining the correlated knowledge. Hence, by solving the objective in (1), the subspace Θ_*a*_ and the decision functions $f_{\theta_{a/t}}^{a/t}$ s can be learned simultaneously. In the sequence, we will focus on designing these components in the general formulation one by one to construct a unified framework.

#### Design of Regression Model

In LMA, we learn a composite source classifier function $f^{a}=P_{a}^{{}^{\circ}}\Theta_{a}$ trained on the EEG features, where *Q*_*a*_ represents the source classifier model, and ° is the function combination operator. We therefore explore to find the best approximation *W*_*t*_ for *f^t^* by leverage *Q*_*a*_ in Θ_*a*_ with the assumption that there exist some commonalities (e.g., discriminative structures) between different domains (Tao et al., 2019). Moreover, it should also maintain the discriminative structure in the original space. To capture the source correlation information, we respectively design the following classification functions for the *a*th source domain in the latent space:


(2) $$f_{\theta }^{a}\left(X^{a}\right)=V_{a}^{T}\phi \left(X^{a}\right)+Q_{a}^{T}\psi_{\theta }\left(X^{a}\right),a=1,2,\ldots ,S,$$

where ϕ:χ→*H*^2^ is a known feature map projecting the *a*th source data from the input space χ into certain reproducing kernel Hilbert space (RKHS) (Nie et al., 2010b) *H*. The other component ψ_θ_ is a parameterized low-dimensional space that aims at encoding the shared structure between each source domain and the target domain. The weight vector *Q*_*a*_ is defined in the projected subspace under the projection ψ_θ_ for the *a*th source, and *V*_*a*_ is the weight matrix defined in the original feature space. With the parametric form in (2), the learned subspace ψ_θ_ can capture the intrinsic structure of source correlation in the MDA problem, which are shared by each source and the target domain. Correspondingly, we also can design the target recognition model: $f^{t}\left(X^{t}\right)=W_{t}^{T}\phi \left(X^{t}\right)$ with the weight matrix *W*_*t*_. We present the empirical kernel map as discussed in Gretton et al. (2009):


$$\begin{array}{c} \psi_{e}:\;\chi \to R^{N},forlinearkernelmapping\\ x\to {K_{\psi }\left(\cdot ,x\right)|}_{x_{1},x_{2},\ldots ,x_{N}}=\left(K_{\psi }\left(x_{1},x\right),\ldots ,K_{\psi }\left(x_{N},x\right)\right),\\ fornonlinearkernelmapping \end{array}.$$

In the following, we will discuss both the linear classification function and the nonlinear (kernel) classification function and integrate them into a unified form.

• *Linear classifier*. We can consider a simple linear form of feature map, where θ = Θ_*a*_ is an *r*×*d* dimensional matrix and ψ_θ_(*x*) = Θ_*a*_ψ(*x*), with a known *d*-dimensional vector function ψ(*x*). Furthermore, following (11), we can take a simple model ϕ(*X^a^*) = ψ(*X^a^*) = *X^a^* into account. We can thereby write the linear classifier as

(3) $$f_{\theta }^{a}\left(X^{a}\right)=V_{a}^{T}X^{a}+Q_{a}^{T}\Theta_{a}X^{a},a=1,2,\ldots ,S.$$

• *Nonlinear classifier.* If we take kernel learning into account and assume that the feature map ϕ(*x*) and ψ(*x*) belong to certain reproducing kernel Hilbert space (RKHS), Eq. (3) therefore can be kernelized. For ψ(*x*), we firstly denote the kernel matrix as *K*_ψ_ = ⟨ψ(*x*_*i*_),ψ(*x*_*j*_)⟩. By using empirical kernel, we have kernel matrix *K^a^* = ϕ(*X^a^*) with ${\left(K^{a}\right)}_{i,j}=\left\langle \phi \left(x_{i}^{a}\right),\phi \left(x_{j}^{a}\right)\right\rangle$, where $x_{i}^{a},x_{j}^{a}\in X^{a}$. Finally, we can let ψ_θ_ = Θ_*a*_ψ_*e*_, where Θ_*a*_ ∈ *R*^*r*×*N*^ is used to transform the empirical kernel vector to an *r*-dimensional space. Let ${\left\{\Psi_{a}^{i}\right\}}_{i=1}^{r}$ denote the weight parameters in the embedded kernel subspace for the *a*th source. Hence, the kernelized decision functions become

(4) $$f_{\theta }^{a}\left(x\right)=\omega_{a}^{T}K^{a}\left(\cdot ,x\right)+\Psi_{a}^{T}\Theta_{a}K^{a}\left(\cdot ,x\right).$$

where ω_*a*_ is the weight coefficients in the original kernel space for the *a*th source.

In order to model the linear case in Eq. (3) and kernel case in Eq. (4) into a unified framework, we introduce


(5) $$T_{a}=\left\{ \begin{array}{c} \begin{array}{cc} V_{a}+Q_{a}^{T}\Theta_{a}, & linear \end{array}\\ \begin{array}{cc} \omega_{a}+\Psi_{a}^{T}\Theta_{a} & kernel \end{array} \end{array}\right..$$

Moreover, in the following, we use two symbols, namely, *W*_*a*_ and *P*_*a*_, where *W*_*a*_ denotes *V*_*a*_ in the linear case and ω_*a*_ in the kernel case, and *P*_*a*_ denotes *Q*_*a*_ in the linear case and Ψ_*a*_ in the kernel case. Then, Eq. (5) becomes $T_{a}=W_{a}+P_{a}^{T}\Theta_{a}$ for both linear and kernel cases, and we can represent the data in linear space and nonlinear space as follows:


(6) $${\bar{X}}^{a/t}=\left\{ \begin{array}{c} \begin{array}{cc} X^{a/t}, & linear \end{array}\\ \begin{array}{cc} K^{a/t}\left(\cdot ,x\right) & ,kernel \end{array} \end{array}\right..$$

In the sequence, we also refer to *X*^*a*/*t*^ as ${\bar{X}}^{a/t}$ if without special denotation for simplicity of expression. As a result, we can formulate the predictors, linear form as in Eq. (4) and nonlinear form as in Eq. (6), in a unified form as depicted in


(7) $$f_{\theta }^{a}\left(X^{a}\right)=T_{a}^{T}X^{a},a=1,2,\ldots ,S$$

We introduce the sparse regression scheme (Shi et al., 2015) by exploiting *l*_2,1_-norm minimization to enhance the robustness against the misclassification. We particularly construct a scaled pseudo label matrix for the target data, i.e., *F* = [*f*_1_, *f*_2_, …, *f_n_t__*] = (*Y^t^*(*Y^t^*)^*T*^)^−1/2^*Y^t^* ∈ ℝ^*n*_*t*_×*c*^, where the scaled pseudo label $f_{i}=y_{i}^{t}$ if $x_{i}^{t}$ is labeled, *f*_*i*_ = 0 otherwise. Therefore, *FF^T^* = *I_n_t__* can be easily derived with additional constraint *F*≥0. We then respectively find the source classifiers trained on *X^a^* (*a* = 1, 2, …, *S*) and the target classifier trained on *X^t^* by minimizing the following loss functions.


(8) $$R\left(f_{\theta_{a}}^{a},Y_{a}\right)={||f_{\theta_{a}}^{a}\left(X^{a}\right)-Y^{a}||}_{2,1}.$$

(9) $$R\left(f^{t},F\right)={||f^{t}\left(X^{t}\right)-F||}_{F}^{2}+\beta \left({||W_{t}||}_{2,1}+tr\left(FLF^{T}\right)\right).$$

where *L* denotes the graph Laplacian matrix induced from the target samples.

Moreover, it is intuitively reasonable that the outputs of *f^a^* s on the target domain are expected to be consistent with those of *f^t^*, which would gradually make *P*_*a*_ and *W*_*t*_ more accurate after lots of iterations. This prediction consistency can be minimized *via* the following residual:


(10) $$\begin{array}{c} Con\left(f^{a},f^{t},X^{t}\right)={||X_{t}^{T}\left(W_{a}+\Theta_{a}P_{a}\right)-F^{a}||}_{F}^{2}\\ +{||X_{t}^{T}W_{t}-F||}_{F}^{2}+{||F^{a}-F||}_{F}^{2}. \end{array}$$

In such a way, *P*_*a*_ and *W*_*t*_ would jointly enhance the target discriminations for the final emotion recognition.

Additionally, based on the parametric form of the decision function $f_{\theta }^{a}$ as in Eq. (7), we introduce the following regularizer:


(11) $$℧\left(f_{\theta }^{a}\right)={||W_{a}||}^{2}+{||T_{a}||}_{2,1}={||T_{a}-\Theta_{a}^{T}P_{a}||}_{F}^{2}+{||T_{a}||}_{2,1},$$

which controls the complexity of each source classifier independently in the original and latent subspaces, respectively.

#### Uncovering Latent Spaces

In this subsection, we will present an effective strategy to capture multiple domain-invariant subspaces to mitigate the domain discrepancy as well as excavate some domain-invariant discriminative information. To this end, we give two main constraints or conditions on uncovering these latent spaces: (1) preserving the within-domain local structures and (2) aligning the inter-domain marginal distribution divergence as well as conditional distribution discrepancy. Following existing feature extraction methods (Tao et al., 2016), we further constrain Θ_*a*_ to be orthogonal on rows, i.e., $\Theta_{a}\Theta_{a}^{T}=I_{r}$, where *r* (typically far less than *d*) is the feature dimensionality in the latent space.

To fulfill the first condition, we construct a locality preserving regularizer to measure the smoothness along the intrinsic discriminative structure of the domain features (Nie et al., 2010b; Shi et al., 2015; Ding et al., 2018c). Specifically, one can construct an undirected graph with a weighted adjacency matrix ∏_*a*_ = [(∏_*a*_)_*i*, *j*_]_*i*, *j* = 1, 2, …, *N*_, which is defined as (Yan et al., 2006):


(12) $${\left(\prod_{a}\right)}_{i,j}=\left\{ \begin{array}{c} \exp\left(-\gamma {||x_{i}-x_{j}||}^{2}\right),\mathrm{if}x_{i}\in \delta_{k}\left(x_{j}\right)\mathrm{or}x_{j}\in \delta_{k}\left(x_{i}\right)\\ \mathrm{and}\mathrm{both}\mathrm{have}\mathrm{the}\mathrm{same}\mathrm{labels}\\ \exp\left(-\frac{\gamma }{{||x_{i}-x_{j}||}^{2}}\right),\mathrm{if}x_{i}\in \delta_{k}\left(x_{j}\right)\mathrm{or}x_{j}\in \delta_{k}\left(x_{i}\right)\\ \mathrm{and}\mathrm{both}\mathrm{have}\mathrm{different}\mathrm{labels},\\ 0,\mathrm{otherwise} \end{array}\right.,$$

where *x*_*i*_, *x*_*j*_ ∈ *X*_*a*_ = [*X^a^*, *X^t^*], δ_*k*_(*x*) denotes the *k* nearest neighbor set of *x*, and the hyper-parameter γ can be empirically computed as ${\bar{\theta }}_{a}\sqrt{c}$ due to the impact of multi-class distribution, where ${\bar{\theta }}_{a}$ is the squared root of the mean norm of *X*_*a*_. Deriving a diagonal matrix Δ_*a*_ from ∏_*a*_ with (Δ_*a*_)_*i*, *i*_ = ∑_*j*_(∏_*a*_)_*i*, *j*_, we then compute the graph Laplacian matrix as *L*_*a*_ = Δ_*a*_−∏_*a*_. Thus, preserving the local geometrical structures of EEG features can be implemented by the following commonly used formulation in the manifold learning (Chen et al., 2013).


(13) $$tr\left(\Theta_{a}^{T}X_{a}L_{a}X_{a}^{T}\Theta_{a}\right).$$

Benefiting from its simplicity and effectiveness, Maximum Mean Discrepancy (Gretton et al., 2009; Pan et al., 2011) has been commonly used to measure the distribution distance between two different domains. Consequently, to meet the second condition, we aim to minimize the MMD in certain optimized RKHS (Gretton et al., 2009). Specifically, the MMD between each domain pair is defined as follows:


(14) $$\begin{array}{c} MMD\left(X^{a},X^{t}\right)\\ =\underset{||\phi ||\leq 1}{\sup}\left(E_{X^{a}∼P}\left[\phi \left(X^{a}\right)\right]-E_{X^{t}∼Q}\left[\phi \left(X^{t}\right)\right]\right)\\ ={||E_{X^{a}∼P}\left[\phi \left(X^{a}\right)\right]-E_{X^{t}∼Q}\left[\phi \left(X^{t}\right)\right]||}_{H} \end{array},$$

The empirical counterpart of the MMD in Eq. (14) can be defined as:


(15) $$MMD\left(X^{a},X^{t}\right)={||\frac{1}{n_{a}}\sum_{x_{i}\in X^{a}}\phi \left(x_{i}\right)-\frac{1}{n_{t}}\sum_{x_{j}\in X^{t}}\phi \left(x_{j}\right)||}_{H},$$

which can recover an asymptotically unbiased estimation of the squared MMD in Eq. (14). Denote the gram matrix ${\tilde{K}}_{a}\left(x_{i},x_{j}\right)=\phi {\left(x_{i}\right)}^{T}\phi \left(x_{j}\right)$ on dataset *X*_*a*_ as


(16) $${\tilde{K}}_{a}=\left[\begin{array}{cc} {\tilde{K}}^{a} & {\tilde{K}}^{at}\\ {\tilde{K}}^{ta} & {\tilde{K}}^{t} \end{array}\right]\in R^{N\times N},$$

where ${\tilde{K}}^{a}$, ${\tilde{K}}^{t}$, and ${\tilde{K}}^{at}$ (or ${\tilde{K}}^{ta}$) are the Gram matrices respectively defined on the source domain, target domain, and cross domain data. Thus, the squared MMD in Eq. (15) can be formulated as


(17) $$MMD\left(X^{a},X^{t}\right)=tr\left({\tilde{K}}_{a}D^{a}\right),$$

where


(18) Dij=a{1na2whenxi,xj∈Xa1nt2whenxi,xj∈Xt-1nantotherwise.

In the sequence, we will take into account the feature map ϕ in linear as well as kernel forms:

λ Linear kernel: ${\tilde{K}}_{a}=X_{a}^{T}\Theta_{a}^{T}\Theta_{a}X_{a}$ if ϕ(*x*) = Θ_*a*_*x*, where *X*_*a*_ = [*x*_1_, *x*_2_, …, *x*_*N*_].

λ Nonlinear kernel: ${\tilde{K}}_{a}=K_{a}^{T}\Theta_{a}^{T}\Theta_{a}K_{a}$ if ϕ(*x*) = Θ_*a*_ψ_*e*_(*x*) = Θ_*a*_*K*_*a*_(⋅, *x*), where ψ_*e*_(*x*) is the empirical kernel map defined in Eq. (4).

Recalling the definition of $\bar{X}$ in Eq. (6), the domain discrepancy criterion defined in (17) can be reformulated:


(19) $$MMD^{2}\left(X^{a},X^{t}\right)=tr\left({\tilde{K}}_{a}D^{a}\right)=tr\left(\Theta_{a}^{T}{\bar{K}}_{a}\Theta_{a}\right),$$

where


(20) $${\bar{K}}_{a}=\left\{ \begin{array}{c} \begin{array}{cc} X_{a}D^{a}X_{a}^{T}, & \mathrm{linear}\mathrm{kernel} \end{array}\\ \begin{array}{cc} K_{a}^{T}D^{a}K_{a}, & \mathrm{nonlinear}\mathrm{kernel} \end{array} \end{array}\right..$$

Note that Eq. (19) could not preserve the local structures of the EEG data from the same class in the latent spaces due to the shortage of semantic alignment. This would significantly deteriorate the learning performance in some cases. To this end, we further address this issue by improving Eq. (19) with the following class distribution matching term:


(21) $$\begin{array}{c} \sum_{l=1}^{c}{||\frac{1}{n_{a}^{l}}\sum_{i=1}^{n_{a}^{l}}\Theta_{a}^{T}\phi \left(x_{i}^{a\left(c\right)}\right)-\frac{1}{n_{t}^{l}}\sum_{j=1}^{n_{t}^{l}}\Theta^{T}\phi \left(x_{j}^{t\left(c\right)}\right)||}_{F}^{2}\\ =\sum_{l=1}^{c}tr\left(\Theta_{a}^{T}{\bar{K}}_{a}^{\left(l\right)}\Theta_{a}\right), \end{array}$$

where


$${\bar{K}}_{a}^{l}=\left\{ \begin{array}{c} \begin{array}{cc} X_{a}^{\left(l\right)}D^{a\left(l\right)}{\left(X_{a}^{\left(l\right)}\right)}^{T}, & linear \end{array}\\ \begin{array}{cc} {\left(K_{a}^{\left(l\right)}\right)}^{T}D^{a\left(l\right)}K_{a}^{\left(l\right)}, & kernel \end{array} \end{array}\right.,$$

$X_{a}^{\left(l\right)}=\left[X^{a\left(l\right)},X^{t\left(l\right)}\right]$, with *X*^*a*(*l*)^ and *X*^*t*(*l*)^ being the datasets of the *l*th class, respectively, from source and target domains, $n_{a}^{l}$ (respectively $n_{t}^{l}$) is the data size of the *l*th class from the *a*th source (respectively target) domain, and the elements of the matrix *D*^*a*(*l*)^ = [*Di*, *j*^*a*(*l*)^] are defined as


(22) $$D_{i,j}^{a\left(l\right)}=\left\{ \begin{array}{c} \begin{array}{ccc} \frac{1}{{\left(n_{a}^{l}\right)}^{2}}, & when & x_{i},x_{j}\in X^{a\left(l\right)} \end{array}\\ \begin{array}{ccc} \frac{1}{{\left(n_{t}^{l}\right)}^{2}}, & when & x_{i},x_{j}\in X^{t\left(l\right)} \end{array}\\ \begin{array}{ccc} \frac{-1}{n_{a}^{l}n_{t}^{l}}, & & otherwise \end{array} \end{array}\right..$$

Equation (21) explicitly forces EEG data from different domains but the same class to be mapped adjacently in the latent spaces. By unifying Eq. (19) and Eq. (21), we can obtain:


(23) $$\sum_{l=0}^{c}tr\left(\Theta_{a}^{T}{\bar{K}}_{a}^{\left(l\right)}\Theta_{a}\right)=tr\left(\Theta_{a}^{T}\left(\sum_{l=0}^{c}{\bar{K}}_{a}^{\left(l\right)}\right)\Theta_{a}\right),$$

where $\bar{K}a^{\left(0\right)}=\bar{K}a$ and *D*^*a*(0)^ = *D^a^*. We further denote $\Lambda^{a}=\sum_{l=0}^{c}{\bar{K}}_{a}^{\left(l\right)}$. By combining Eq. (13) and Eq. (23), we can attempt to uncover a latent space by minimizing the following formulation:


(24) $$dist_{\Theta_{a}}\left(X^{a},X^{t}\right)=tr\left(\Theta_{a}^{T}\left(X_{a}L_{a}X_{a}^{T}+\Lambda^{a}\right)\Theta_{a}\right)\begin{array}{cc} s.t. & \Theta_{a}^{T}\Theta_{a}=I_{r} \end{array}.$$

#### Sharing Source Discriminative Structure

While each source model is learned in different latent space from each other, one still can presume that these source models might be correlated due to the correlation of source EEG signals in the model level (Tao et al., 2016). These correlated discriminative structures can be encoded by a low rank matrix of all source models, thus transferring the source knowledge from each other. For its simplicity of computation, the following trace norm of the matrix *P* = [*P*_1_, *P*_2_, …, *P*_*S*_], which is a surrogate of the rank minimization, can be adopted for correlating the source models.


(25) $$\Omega \left(f_{\theta }^{a}\right)={||P||}_{*}=tr\left({\left(PP^{T}\right)}^{\frac{1}{2}}\right),$$

### Overall Formulation

Combining the above formulations respectively defined in Eqs (8) to (11) and Eqs (24) and (25) together, we have the following objective function:


(26) $$\begin{array}{c} ℜ=\underset{\begin{array}{ccc} P_{a},W^{t},T_{a},F_{a}, & & \\ F,W^{t},\vartheta ,\eta ,\Theta & & \end{array}}{\arg\min}\sum_{a=1}^{S}[\vartheta_{a}^{q_{1}}|Y_{a}-X_{a}^{T}T_{a}{|}_{2,1}\\ +\alpha \left(|T_{a}{|}_{2,1}+|T_{a}-\Theta_{a}P_{a}{|}_{F}^{2}\right)+\eta_{a}^{q_{2}}|F_{a}-X_{t}^{T}T_{a}{|}_{2,1}^{2}]\\ +\sum_{a=1}^{S}\eta_{a}^{q_{2}}tr\left(\Theta_{a}^{T}C_{a}\Theta_{a}\right)+\sum_{a=1}^{S}\eta_{a}^{q_{2}}{\left|F_{a}-F\right|}_{F}^{2}\\ +{\left|X_{t}^{T}W_{t}-F\right|}_{F}^{2}+\beta \left({\left|W_{t}\right|}_{2,1}+tr\left(FLF^{T}\right)\right)+\frac{\lambda }{2}{\left|P\right|}_{*}\\ s.t.\sum_{a=1}^{S}\vartheta_{a}=\sum_{a=1}^{S}\eta_{a}=1,\Theta_{a}^{T}\Theta_{a}=I_{r} \end{array},$$

where *C*_*a*_ = $X_{a}L_{a}X_{a}^{T}+\Lambda^{a}$, ϑ = [ϑ_1_, …,ϑ_*a*_]^*T*^ is the weight vector to jointly combine all source regression loss, α, β, and λ are three regularization parameters, *q*_1_, *q*_2_ > 1 are two tunable parameters for avoiding trivial solution, and the tunable vector η = [η_1_,η_2_, …,η_*S*_] denotes the adaptation degrees of different sources. The *l*_2,1_-norm regularization added on projection matrix *P*_*a*_ forces most of the rows in *P*_*a*_ (*a* = 1, …, *S*) to shrink to zero, thus performing feature selection on original data.

### Emotion Recognition

After the best model parameters have been pursued, the source and target classifiers can be applied to recognize the emotion level of each probe EEG data. Specifically, we linearly fuse two recognition results on the probe data, i.e., $f^{s}\left(X^{t}\right)=\sum_{a=1}^{S}\vartheta_{a}{\left(X^{t}\right)}^{T}\left(\Theta_{a}P_{a}+W_{a}\right)$ obtained from source models, and *f^t^*(*X^t^*) = (*X^t^*)^*T*^ *W^t^* predicted from the target model, as the final prediction value. That is, the following combination function can be exploited for recognizing the emotion level of the given test data $x_{i}^{t}$:


$$j=\arg\max_{j}{\left(y_{i}^{t}=\delta f^{s}\left(x_{i}^{t}\right)+\left(1-\delta \right)f^{t}\left(x_{i}^{t}\right)\right)}_{j},$$

where δ is a trade-off parameter, tuned from [0,1]. In the experimental setting, we empirically set δ = 0.5 for initialization, followed by the evaluation of its impact on performance with different values of it.

## Algorithm

In this section, an alternately iterative procedure is adopted to optimize the objective function in Eq. (26), which is followed by an overall algorithm.

### Optimization

In terms of the definition in Nie et al. (2010a), we can derive ${\left|\tilde{T}\right|}_{2,1}=2tr\left({\tilde{T}}^{T}Q\tilde{T}\right)$, where *Q* is a diagonal matrix with the *i*th diagonal element $Q_{ii}=\frac{1}{2{||{\tilde{T}}_{i,:}||}_{2}}$. Hence, we can further transform Eq. (26) into Eq. (27):


(27) $$\begin{array}{c} ℜ=\underset{\begin{array}{c} P_{a},W_{t},T_{a},F_{a},\\ F,\vartheta ,\eta ,\Theta \end{array}}{\arg\min}\sum_{a=1}^{S}\left(\begin{array}{ccc} \vartheta_{a}^{q_{1}}tr\left({\tilde{T}}_{a}^{T}Z_{a}{\tilde{T}}_{a}\right)+\alpha tr(T_{a}^{T}G_{a}T_{a} & & \\ +|T_{a}-\Theta_{a}P_{a}{|}_{F}^{2}) & & \\ +\eta_{a}^{q_{2}}(tr\left(\Theta_{a}^{T}C_{a}\Theta_{a}\right)+|F_{a}-F{|}_{F}^{2} & & \\ +tr\left(Q_{a}^{T}{\tilde{Z}}_{a}Q_{a}\right)) & & \end{array}\right)\\ +\frac{\lambda }{2}tr\left(P^{T}{\left(PP^{T}\right)}^{-\frac{1}{2}}P\right)+{\left|X_{t}^{T}W_{t}-F\right|}_{F}^{2}+\beta \left({\left|W_{t}\right|}_{2,1}+tr\left(FLF^{T}\right)\right)\\ s.t.\sum_{a=1}^{S}\vartheta_{a}=\sum_{a=1}^{S}\eta_{a}=1,\Theta_{a}^{T}\Theta_{a}=I_{r} \end{array},$$

where ${\tilde{T}}_{a}=\left(Y_{a}-X_{a}^{T}T_{a}\right)$ and $Q_{a}=F_{a}-X_{t}^{T}T_{a}$.

By solving the derivative of Eq. (27) w.r.t. *W*_*t*_ and letting it equal to zero:


(28) $$\frac{\partial ℜ}{\partial W_{t}}=0\Rightarrow W_{t}=\Delta^{-1}X_{t}F,$$

where $\Delta =X_{t}X_{t}^{T}+\beta \tilde{V}$, and $\tilde{V}$ is a diagonal matrix with the *k*th diagonal element being ${\left(\tilde{V}\right)}_{kk}=\frac{1}{2{||{\left(W_{t}\right)}_{k,:}||}_{2}}$. Substituting *W*_*t*_ in Eq. (27) with Eq. (28), we have:


(29) ℜ=arg⁡minPa,Ta,Fa,F,ϑ,η,Θ∑a=1S(ϑaq1tr(T~aTZaT~a)+α(tr(TaTGaTa)+|Ta-ΘaPa|F2)+|Fa-F|F2+ηaq2(tr(ΘaTCaΘa)+tr((Fa-XtTTa)Z~aT(Fa-XtTTa))))+λ2tr(PT(PPT)-12P)+βtr(FΔ~FT)s.t.∑a=1Sϑa=∑a=1Sηa=1,ΘaTΘa=Ir,

where $\tilde{\Delta }=\beta L+\left(X_{t}^{T}\Delta^{-1}X_{t}-I\right)+\beta X_{t}^{T}\Delta^{-1}\tilde{V}\Delta^{-1}X_{t}$. By solving the derivative of Eq. (29) w.r.t. *F*_*a*_ and letting it equal to zero, we have:


(30) $$\begin{array}{c} \frac{\partial ℜ}{\partial F_{a}}=2{\tilde{Z}}_{a}F_{a}-2{\tilde{Z}}_{a}X_{t}^{T}T_{a}+2F_{a}-2F=0\\ \Rightarrow F_{a}=S_{a}^{-1}\left(F+{\tilde{Z}}_{a}X_{t}^{T}T_{a}\right) \end{array},$$

where $S_{a}={\tilde{Z}}_{a}+I$. Plugging *F*_*a*_ in Eq. (30) into Eq. (29), we can get:


(31) $$\begin{array}{c} \eta_{a}^{r}tr\left({\left(S_{a}^{-1}F+\left(S_{a}^{-1}{\tilde{Z}}_{a}X_{t}^{T}-X_{t}^{T}\right)T_{a}\right)}^{T}{\tilde{Z}}_{a}\left(S_{a}^{-1}F+\left(S_{a}^{-1}{\tilde{Z}}_{a}X_{t}^{T}-X_{t}^{T}\right)T_{a}\right)\right)\\ +\eta_{a}^{r}{||\left(S_{a}^{-1}-I\right)F+S_{a}^{-1}{\tilde{Z}}_{a}X_{t}^{T}T_{a}||}_{F}^{2}+\beta tr\left(F\tilde{\Delta }F^{T}\right) \end{array}.$$

By solving the derivative of Eq. (31) w.r.t. *F* and letting it equal to 0, we obtain:


(32) $$F=G_{a}^{-1}D_{a}T_{a},$$

where


(33) $$\left\{ \begin{array}{c} G_{a}=\eta_{a}^{q_{2}}S_{a}^{-1}{\tilde{Z}}_{v}S_{a}^{-1}+\eta_{a}^{q_{2}}{\left(S_{a}^{-1}-I\right)}^{T}\left(S_{a}^{-1}-I\right)+\tilde{\Delta }\\ D_{a}=\eta_{a}^{q_{2}}\left(S_{a}^{-1}-I\right)S_{a}^{-1}{\tilde{Z}}_{a}X_{t}^{T}+\eta_{a}^{q_{2}}S_{a}^{-1}{\tilde{Z}}_{a}\left(S_{a}^{-1}{\tilde{Z}}_{a}-I\right)X_{t}^{T} \end{array}\right.,$$

By replacing *F* and *F*_*a*_ in Eq. (29) with those in Eqs (30) and (32), respectively, and solving the derivative of Eq. (29) with reference to *T*_*a*_ and equaling to zero, we then get:


(34) $$T_{a}=H_{a}^{-1}\left(\vartheta_{a}^{q_{1}}X_{a}Z_{a}Y_{a}+\alpha \Theta_{a}P_{a}\right),$$

where:


(35) $$\begin{array}{ccc} H_{a}=\vartheta_{a}^{q_{1}}X_{a}Z_{a}X_{a}^{T}+\alpha \left(G_{a}+I\right)+\eta_{a}^{q_{2}}(\left(S_{a}^{-1}-I\right)G_{a}^{-1}D_{a} & & \\ +S_{a}^{-1}{\tilde{Z}}_{a}X_{t}^{T}){}^{T}(\left(S_{a}^{-1}-I\right)G_{a}^{-1}D_{a}+S_{a}^{-1}{\tilde{Z}}_{a}X_{t}^{T}) & & \\ +\beta D_{a}^{T}G_{a}^{-1}\tilde{\Delta }G_{a}^{-1}D_{a}+\eta_{a}^{q_{2}}{\left(S_{a}^{-1}G_{v}^{-1}D_{v}+S_{a}^{-1}{\tilde{Z}}_{a}X_{t}^{T}-X_{t}^{T}\right)}^{T} & & \\ {\tilde{Z}}_{a}\left(S_{a}^{-1}G_{v}^{-1}D_{v}T_{v}+S_{a}^{-1}{\tilde{Z}}_{a}X_{t}^{T}-X_{t}^{T}\right) & & \end{array}.$$

Let $U_{a}=\frac{1}{2}{\left(PP^{T}\right)}^{-\frac{1}{2}}$ and by replacing *T*_*a*_ in Eq. (29) with Eq. (34), and solving the derivative of Eq. (29) in reference to *P*_*a*_ and equaling to zero, we then get:


(36) $$P_{a}=-{\left(\Theta_{a}^{T}O_{a}\Theta_{a}+\lambda U_{a}\right)}^{-1}M_{a}^{T}\Theta_{a},$$

where


(36-1) $$O_{a}=\left(\begin{array}{cccc} \vartheta_{a}^{q_{1}}\alpha^{2}H_{a}^{-1}X_{a}Z_{a}X_{a}^{T}H_{a}^{-1}+\alpha^{3}H_{a}^{-1}G_{a}H_{a}^{-1} & & & \\ +\alpha {\left(\alpha H_{a}^{-1}-I\right)}^{T}\left(\alpha H_{a}^{-1}-I\right) & & & \\ +\eta_{a}^{q_{2}}\alpha^{2}H_{a}^{-1}{\left(\left(S_{a}^{-1}-I\right)G_{a}^{-1}D_{a}+S_{a}^{-1}{\tilde{Z}}_{a}X_{t}^{T}\right)}^{T} & & & \\ \left(\left(S_{a}^{-1}-I\right)G_{a}^{-1}D_{a}+S_{a}^{-1}{\tilde{Z}}_{a}X_{t}^{T}\right)H_{a}^{-1} & & & \\ +\eta_{a}^{q_{2}}\alpha^{2}H_{a}^{-1}{\left(S_{a}^{-1}G_{v}^{-1}D_{v}+S_{a}^{-1}{\tilde{Z}}_{a}X_{t}^{T}-X_{t}^{T}\right)}^{T} & & & \\ {\tilde{Z}}_{a}\left(S_{a}^{-1}G_{v}^{-1}D_{v}+S_{a}^{-1}{\tilde{Z}}_{a}X_{t}^{T}-X_{t}^{T}\right)H_{a}^{-1} & & & \\ +\beta \alpha^{2}H_{a}^{-1}D_{a}^{T}G_{a}^{-1}\tilde{\Delta }G_{a}^{-1}D_{a}H_{a}^{-1} & & & \end{array}\right),$$

(36-2) $$M_{a}=\left(\begin{array}{cccc} \vartheta_{a}^{q_{1}}\alpha H_{a}^{-1}X_{a}Z_{a}\left(\vartheta_{a}^{q}X_{a}^{T}H_{a}^{-1}X_{a}Z_{a}Y_{a}-Y\right)+\alpha^{2}\vartheta_{a}^{q_{1}} & & & \\ H_{a}^{-1}G_{a}H_{a}^{-1}X_{a}Z_{a}Y_{a}+\vartheta_{a}^{q_{1}}\left(\alpha H_{a}^{-1}-I\right)H_{a}^{-1}X_{a}Z_{a}Y_{a} & & & \\ +\eta_{a}^{r}\vartheta_{a}^{q_{1}}\alpha H_{a}^{-1}{\left|\left(S_{a}^{-1}-I\right)G_{a}^{-1}D_{a}+S_{a}^{-1}{\tilde{Z}}_{a}X_{t}^{T}\right|}_{F}^{2}H_{a}^{-1} & & & \\ X_{a}Z_{a}Y_{a}+\beta \alpha \vartheta_{a}^{q_{1}}H_{a}^{-1}D_{a}^{T}G_{a}^{-1}\tilde{\Delta }G_{a}^{-1}D_{a}H_{a}^{-1}X_{a}Z_{a}Y_{a} & & & \\ +\eta_{a}^{r}\vartheta_{a}^{q_{1}}\alpha H_{a}^{-1}{\left(S_{a}^{-1}G_{v}^{-1}D_{v}+S_{a}^{-1}{\tilde{Z}}_{a}X_{t}^{T}-X_{t}^{T}\right)}^{T}{\tilde{Z}}_{a} & & & \\ \left(S_{a}^{-1}G_{v}^{-1}D_{v}+S_{a}^{-1}{\tilde{Z}}_{a}X_{t}^{T}-X_{t}^{T}\right)H_{a}^{-1}X_{a}Z_{a}Y_{a} & & & \end{array}\right).$$

By substituting the optimal solution of the updated variables in Eqs (30), (32), (34), and (36) into Eq. (29) by mathematical calculation with the constraints $\Theta_{a}^{T}\Theta_{a}=I_{r}$, we then can get the following objective function in reference to Θ_*a*_:


(37) $$ℜ\left(\Theta_{a}\right)=\min_{\Theta_{a}}tr\left(\Theta_{a}^{T}\left(\eta_{a}^{q_{2}}C_{a}-M_{a}{\left(\Theta_{a}^{T}O_{a}\Theta_{a}+\lambda U_{a}\right)}^{-1}M_{a}^{T}\right)\Theta_{a}\right),$$

which is equivalent to the following objective:


(38) $$ℜ\left(\Theta_{a}\right)=\max_{\Theta_{a}^{T}\Theta_{a}=I_{r}}tr\left(\Theta_{a}^{T}R_{a}\Theta_{a}\right),$$

where $R_{a}=M_{a}{\left(\Theta_{a}^{T}O_{a}\Theta_{a}+\lambda U_{a}\right)}^{-1}M_{a}^{T}-\eta_{a}^{q_{2}}C_{a}$. According to Li et al. (2015), Θ_*a*_ can be relaxedly obtained by the Eigen-decomposition of *R*_*a*_.

Lastly, we respectively optimize ϑ_*a*_ and η_*a*_ by fixing other variables. In this situation, the objective in Eq. (29) by preserving ϑ_*a*_ changes to the following problem:


(39) $$\min_{\vartheta_{a}\geq 0,\vartheta_{a}^{T}1=1}\sum_{a=1}^{S}\vartheta_{a}^{q}tr\left({\tilde{T}}_{a}^{T}Z_{a}{\tilde{T}}_{a}\right),$$

Let $g_{a}=tr\left({\tilde{T}}_{a}^{T}Z_{a}{\tilde{T}}_{a}\right)$, the Lagrange function of Eq. (39) is


(40) $$ℑ\left(\vartheta_{a},\varphi \right)=\sum_{a=1}^{S}\vartheta_{a}^{q_{1}}g_{a}-\varphi \left(\sum_{a=1}^{S}\vartheta_{a}-1\right),$$

Let the derivative of ℑ⁡(ϑ_*a*_,φ) with respect to ϑ_*a*_ be equivalent to 0 and we can obtain:


(41) $$\vartheta_{a}={\left(\varphi /\left(q_{1}g_{a}\right)\right)}^{\frac{1}{q_{1}-1}},$$

Substituting Eq. (41) into the constraint $\sum_{a=1}^{S}\vartheta_{a}=1$, we obtain


(42) $$\vartheta_{a}={\left(g_{a}\right)}^{1/\left(1-q_{1}\right)}/\sum_{a=1}^{S}{\left(g_{a}\right)}^{1/\left(1-q_{1}\right)},$$

With the same deduction with that of ϑ_*a*_, we also get the following optimal solution of η_*a*_:


(43) $$\eta_{a}={\left(h_{a}\right)}^{1/\left(1-q_{2}\right)}/\sum_{a=1}^{S}{\left(h_{a}\right)}^{1/\left(1-q_{2}\right)},$$

where $h_{a}=tr\left(\Theta_{a}^{T}C_{a}\Theta_{a}\right)+||F_{a}-F|{|}_{F}^{2}+tr({\left(F_{a}-X_{t}^{T}T_{a}\right)}^{T}$${\tilde{Z}}_{a}\left(F_{a}-X_{t}^{T}T_{a}\right))$.

### Overall Algorithm

An overall optimization process of LMA can be outlined in the Algorithm 1. Following the same strategy in Zhang et al. (2019b), we employ a window-based breaking criterion to better achieve the convergence state of the algorithm. In terms of this strategy, we denote by ℏ = 6 the window size and compute ς = |*MaxObj*_*itr*_−*MinObj*_*itr*_|/*MaxObj*_*itr*_ in *itr*−*th*iteration, where *Obj*_*itr*_ = {*Obj*_*itr*−ℏ + 1_, …, *Obj*_*itr*_} represents the set of historical target values in the window. While ς < ε = 10^−5^, our algorithm will stop the iteration.

**Table:** 

Algorithm 1: Multi-source adaptation learning.
**Input:** Source datasets ${\left\{X_{i}^{s}\right\}}_{i=1}^{S}$, Laplacian matrices ${\left\{L_{i}\right\}}_{i=1}^{S}$, target dataset *X^t^*, and parameters *α*, *β*, and *λ*, the maximal iteration number *ℓ*. **Output:** Converged projection matrices ${\left\{P_{i}\right\}}_{i=1}^{S}$, ${\left\{\Theta_{i}\right\}}_{i=1}^{S}$, and matrices ${\left\{F_{i}\right\}}_{i=1}^{S}$ and *W*_*t*_. **Initialization:** Set *itr* = 0, and initialize Θ_*a*_ = *I*_*r*_ and ${\left\{P_{a}^{itr}\right\}}_{a=1}^{S}$ randomly. Let $P^{itr}=\left[P_{1}^{itr},\ldots ,P_{S}^{itr}\right]$; **1: for *a = 1* to *S* do** { 1) Compute matrix $D_{itr}^{a}$ and $D_{itr}^{a\left(l\right)}$, and ${\bar{K}}_{a}^{itr}$ and ${\bar{K}}_{a\left(l\right)}^{itr}$ with empirical kernel mapping, thus computing $\Lambda^{a}=\sum_{l=0}^{c}{\bar{K}}_{a}^{\left(l\right)}$, *l* = 1, …, *c* and Compute $\vartheta_{a}=\frac{tr\left(X_{a}L_{a}X_{a}^{T}+\Lambda^{a}\right))}{\sum_{a=1}^{S}tr\left(X_{a}L_{a}X_{a}^{T}+\Lambda^{a}\right))}$; 2) Initialize *T*_*a*_ = Θ_*a*_*P*_*a*_ and $F^{itr}=\sum_{a=1}^{S}\vartheta_{a}{\left(X^{t}\right)}^{T}\Theta_{a}P_{a}$; } **2: repeat** { 3) Compute $W_{t}^{itr}$ by Eq. (28) 4) Compute the matrix ${\tilde{V}}^{itr}$ with ${\left(\tilde{V}\right)}_{kk}=\frac{1}{2{||{\left(W_{t}^{itr}\right)}_{k,:}||}_{2}}$; 5) set *a=1*; **repeat** { 6) Compute the diagonal matrix $Z_{a}^{itr}$, $G_{a}^{itr}$, and ${\tilde{Z}}_{a}^{itr}$; 7) Compute $S_{a}^{itr}={\tilde{Z}}_{a}^{itr}+I$; 8) Compute Θ_*a*_ according to Eq. (38) and then compute $\eta_{a}^{itr}$ according to Eq. (43); 9) Compute $F_{a}^{itr}={\left(S_{a}^{itr}\right)}^{-1}\left(F^{itr}+{\tilde{Z}}_{a}^{itr}X_{t}^{T}T_{a}^{itr}\right)$ by (30); 10) Compute *F^itr^* by (32) after computing $G_{a}^{itr}$ and $D_{a}^{itr}$ by (33); 10) Compute $T_{a}^{itr}$ by (34) with (35); 11) Compute $P_{a}^{itr}$ by (36) after computing (36-1) and (36-2); 12) Compute $\vartheta_{a}^{itr}$ according to Eq. (42); 13) Compute the matrix $R_{a}^{itr}=M_{a}^{itr}{\left({\left(\Theta_{a}^{itr}\right)}^{T}O_{a}^{itr}\Theta_{a}^{itr}+\lambda U_{a}^{itr}\right)}^{-1}{\left(M_{a}^{itr}\right)}^{T}-{\left(\eta_{a}^{itr}\right)}^{q_{2}}C_{a}^{itr}$; 14) *a* = *a* + 1; }**until *a* > *S*** 7) Update $P_{a}^{itr+1}=P_{a}^{itr}$, thus $\Theta_{a}^{itr+1}=\Theta_{a}^{itr}$s.t.*a* = 1,.., *S*; 8) Update $F_{a}^{itr+1}=F_{a}^{itr}$ according to (30) s.t.*a* = 1,.., *S*; 9) Update $\vartheta_{i}^{itr+1}$ according to (42) s.t.*a* = 1,.., *S*; 10) Update $\eta_{i}^{itr+1}$ according to (43) s.t.*a* = 1,.., *S*; 11) Update *F*^*itr* + 1^ by (32), thus $W_{t}^{itr+1}$ according to (28); 12) Let *itr* = *itr* + 1; }**until *itr* > ℓ or ς < 10^−5^** **3: return ${\left\{P_{a}\right\}}_{a=1}^{S}$,** ${\left\{\Theta_{a}\right\}}_{a=1}^{S}$, *W*_*t*_, *F*** and ${\left\{F_{i}\right\}}_{a=1}^{S}$.**

In terms of the proof in Nie et al. (2010a), the convergence of the iterative procedures in Algorithm 1 can be guaranteed by the following theorem.

**Theorem 1** (Tao and Dan, 2021). The objective value in Eq. (29) would steadily decline after several iterations by Algorithm 1, thus finally converging to the optimum.

## Experimental Evaluation

In this part, we comprehensively compare the proposed method with several state of the arts on two widely used benchmark databases including SEED (Zheng and Lu, 2015) and DEAP (Koelstra et al., 2012) for EEG-based emotion recognition (Mansour et al., 2009).

### Databases

According to Zhong et al. (2020) and Lan et al. (2018), there exist certain significant differences between SEED and DEAP since they can be generated by different subjects, sessions, EEG devices, experimental schemes, and emotional stimuli. Detailed information about these two datasets can be viewed in Lan et al. (2018). In the following experiments, we adopt the differential entropy (DE) (Lan et al., 2018; Zhong et al., 2020) as the data feature in emotion recognition, which has also been widely used in the preceding literatures (Shi et al., 2013; Zhang et al., 2015; Chai et al., 2016; Zheng and Lu, 2016; Chai et al., 2017; Lan et al., 2018; Zhong et al., 2020) for DA emotion recognition.

### Baselines and Setting

We will systematically compare our method with such state of the arts as FSSL, an effective feature selection method without DA, FastDAM (Duan et al., 2012a), Multi-KT (Tommasi et al., 2014) with *l*_2_-norm constraint on *p*, A-SVM (Yang et al., 2007), and DSM (Duan et al., 2012a). Since existing deep DA frameworks have achieved many inspiring results on emotion recognition as well as visual recognition, we also additionally present comparisons with several deep (CNN-based) DA methods with deep features: DAN (Long et al., 2015), ReverseGrad (Ganin and Lempitsky, 2015), and MultiDIAL (Carlucci et al., 2020) based on AlexNet, SDDA (Ding et al., 2018a), and CCSA (Motiian et al., 2017), a unified framework of supervised DA and generalization with deep models.

In our multi-source adaptation settings, for the baselines FSSL and A-SVM, we just equally fuse all prediction values of the base classifiers respectively obtained from each source domain^3^.

In our method, LMA, there exist three vital parameters, i.e., λ, α, and β, that need to be tuned. In the community of machine learning, how to jointly search the best parameter values is still a yet unaddressed open issue. Consequently, we empirically choose these parameters using the grid search strategy also adopted in our previous work (Tao et al., 2019). Specifically, we fine-tune the values of λ, α, and β from the grid range {10^−4^,10^−3^, …,10^3^,10^4^} in a heuristic way. Additionally, we also empirically set *q*_1_ = *q*_2_ = 2 for preventing the trivial solution in terms of the conclusion reported in Hou et al. (2017). Finally, we search the nearest neighbor number *k* from the set {35,10,15,17}, which is also adopted in FSSL. In Algorithm 1, we pre-set the maximum iteration number τ = 100.

Through our experiments, we adopt the RBF kernel function, i.e., *K*_*i*, *j*_ = exp (−σ||*x*_*i*_−*x*_*j*_||^2^), for all nonlinear methods, where σ is equal to 1/*d*. In FastDAM, we operate the same practice in Duan et al. (2012a) and set $\gamma_{i}=\frac{\exp\left(-\delta Dist\left(X_{i}^{s},X\right)\right)}{\sum_{i}\exp\left(-\delta Dist\left(X_{i}^{s},X\right)\right)}$ (*i* = 1, …, *S*.), where δ = 100.

### Cross-Subject Emotion Recognition

Note that different subjects even from the same dataset still have different EEG feature distributions due to the individual characteristics. We therefore practice the so-called leave-one-out cross-validation strategy conducted also in Lan et al. (2018) to evaluate the emotion recognition performance. That is, one subject remains to be the target domain, and others from the dataset are constructed as multiple sources. In this multi-source scenario, we follow the same setting as Tao and Dan (2021) to evaluate our method compared with other state of the arts on SEED and DEAP, respectively.

#### Performance Evaluation

**FIGURE 2:**
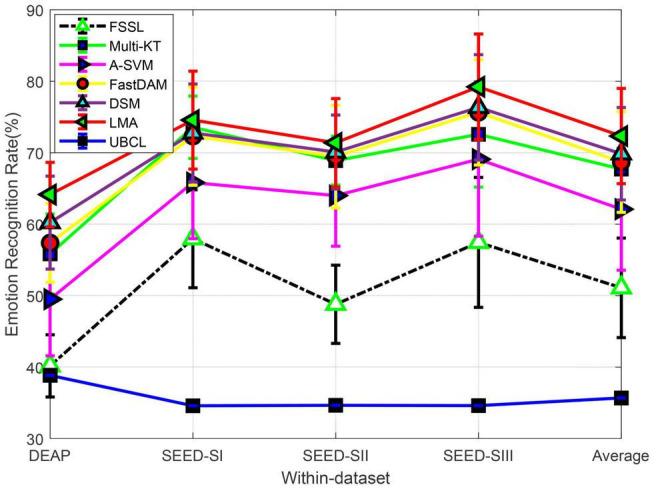
Domain adaptation emotion recognition on within-dataset (SI: Session I, SII: Session II, SIII: Session III).

We plot in Figure 2 the recognition performance of LMA compared with the baselines on two benchmark datasets. The final obtained upper bound of chance level (UBCL) with 95% confidence interval is also recorded in Figure 2. It is well known that the theoretical performance (or chance level) (about 33.33 %) of the random prediction could be achieved approximately by the real chance level if the size of training data approached infinity (Lan et al., 2018). When there are finite samples, we obtain the empirical chance level by repeating the trials with the samples in question equipped with randomized class labels (Lan et al., 2018).

From Figure 2, we can observe that the mean performance (40.16%) of FSSL on DEAP is very close to the random prediction. While it has significantly exceeded UBCL at a 5% significance level, the relatively worse performance of FSSL still indicates the imperative importance of DA in cross-subject emotion recognition due to the substantial distribution divergence between different subjects. This importance has been witnessed by almost all baseline adaptation methods, which have yielded better performance than FSSL in all cross-subject settings. Specifically, our method, LMA, undoubtedly obtains the best recognition accuracy (about 25.14% gains over FSSL), which is closely followed by DSM. While all DA as well as our method, LMA, achieved on DEAP obvious improvement over FSSL with respect to *t*-test with *p*-value > 0.05, the mean recognition performance of these methods is yet not satisfied so far due to the complexity and difference among all subjects.

The no-adaptation method FSSL touched on SEED an average accuracy of 53.78% on three sessions from SEED, which significantly outperformed UBCL. Those multi-source adaptation methods including our method, LMA, unsurprisingly achieved more accuracy gains than the no-adaptation method on SEED. We can still observe that our method, LMA, demonstrates the best performance on SEED by upgrading the average accuracy with 75.47% w.r.t. *t*-test with *p*-value > 0.05. An interesting observation is that all methods work better on SEED than DEAP, which has also been reported in Lan et al. (2018) and Tao and Dan. (2021). The reason for this phenomenon might be that the larger distribution discrepancy between different subjects from DEAP prevented boosting performance in these methods (Mansour et al., 2009; Lan et al., 2018).

#### Multiple Kernel Selection

As well known, the choice of kernel is a challenging issue in the kernel learning method. Recently, multiple kernel learning (MKL) has been effectively proposed for conquering this choice issue existing in single kernel learning methods. Consequently, we also evaluate the performance boost in our method by using MKL (called as MKLMA for short) for each source domain. To this end, the first step is to construct a new space spanned by multiple kernel mapping features. We firstly denote by ${\left\{\phi_{a}\right\}}_{a=1}^{℧}$ an empirical kernel function set, which respectively projects *X*_*a*_ into ℧ different spaces. Then, an orthogonally integrated space can be constructed by concatenating these ℧ spaces. We denote the mapping features in this final space by $\tilde{\phi }\left(x_{i}\right)={\left[\phi_{1}{\left(x_{i}\right)}^{T},\phi_{2}{\left(x_{i}\right)}^{T},\ldots ,\phi_{℧}{\left(x_{i}\right)}^{T}\right]}^{T}\in {\mathbb{R}}^{℧n_{a}}$, where *x*_*i*_ ∈ *X*_*a*_. Correspondingly, the kernel matrix in this final space can be easily deduced as $K_{new}=\left[{\tilde{K}}_{1};{\tilde{K}}_{2};\ldots ;{\tilde{K}}_{℧}\right]$, where ${\tilde{K}}_{i}$ is the *i*th kernel matrix from the ℧ feature spaces. Aiming to exploit the multiple kernel spaces, we therefore employ four kernel mapping functions including the above-used Gaussian kernel. The other additionally employed kernels are inverse square distance kernel function, Laplacian kernel function, and inverse distance kernel function, respectively, denoted as *K*_*ij*_ = 1/(1 + σ||*x*_*i*_−*x*_*j*_||^2^), $K_{ij}=\exp\left(-\sqrt{\sigma }||x_{i}-x_{j}||\right)$, and $K_{ij}=1/\left(1+\sqrt{\sigma }||x_{i}-x_{j}||\right)$.

**FIGURE 3:**
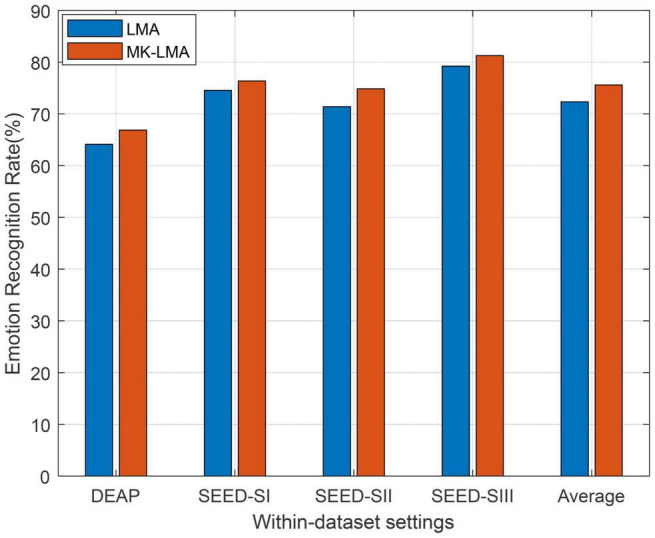
Domain adaptation emotion recognition on within-dataset with multi-kernel learning (SI: Session I, SII: Session II, SIII: Session III).

The observation from Figure 3, in which MKLMA significantly outperforms LMA, justifies that our LMA with MKL can further boost the recognition performance on DEAP and SEED. This also proves the importance of kernel choice in those kernel-based learning models.

### Cross-Dataset Emotion Recognition

#### Single-Source Adaptation

In this subsection, we will demonstrate the consistent robustness of LMA by evaluating its performance in several cross-dataset settings, which is more challenging than the cross-subject adaptation due to the intrinsic difference between datasets. For the scenario of cross-dataset adaptation, we specially design several different cross-dataset strategies by splitting the training set and test set, respectively, in terms of their EEG instruments and emotional stimuli sources, thus making up six cases, i.e., *DEAP*→*SI*, *DEAP*→*SII*, *DEAP*→*SIII*, *SI*→*DEAP*, *SEED II*→*DEAP*, and *SIII*→*DEAP*, where A→B denotes the adaptation from the dataset A to the dataset B, and SI, SII, and SIII are respectively denoted as the dataset of session I, session II, and session III from the database SEED.

A representative hypothesis used in DA is that the feature space of both source and target domains should be the same. Following this assumption, we employ in this part only 32 channels shared between SEED and DEAP to construct a common feature space with 160 dimensions for both domain datasets. In the first three trials, we sample 2,520 samples as the source from DEAP and 2,775 samples as the target from three different sessions (SI, SII, and SIII) in SEED. We evaluate each subject with respect to recognition accuracy in each session and then record the final mean results over 15 subjects from SEED. In the other trials, we resample 41,625 source samples from SEED and 180 target samples from DEAP. We also record the mean recognition accuracy of each subject in DEAP over 14 subjects. For the limitation of memory, 10% of the source data (4,162 samples) is randomly sampled as the actual training samples (Shi et al., 2013; Zheng and Lu, 2015, 2016; Chai et al., 2016, 2017; Lan et al., 2018; Zhong et al., 2020).

**FIGURE 4:**
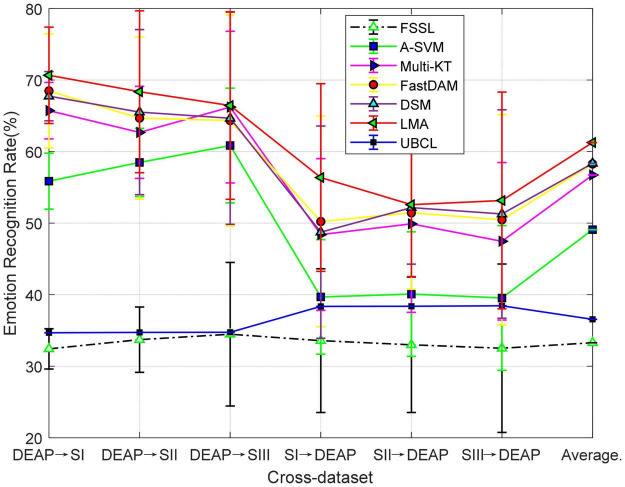
Domain adaptation emotion recognition on cross-dataset (SI: Session I, SII: Session II, SIII: Session III).

The mean recognition results on six cross-datasets are plotted in Figure 4. We can observe from these results that the performance of FSSL is almost near the random guess in that it is slightly inferior to UBCL with about 95% confidence interval. Besides, as observed from the results, the mean performance of each method is slightly worse on cross-dataset than within-dataset. This confirms the larger distribution gaps between two datasets than within-dataset. The advantage of DA would be reflected in this situation since DA could potentially relieve the distribution issue in the cross-dataset applications, which can also be justified by the observation from Figure 4, where all DA methods outperform the no-adaptation one. While Multi-KT and FastDAM occasionally obtain the best performance in some settings, our method, LMA, still contributes the best performance in most cases.

#### Multi-Source Adaptation

**FIGURE 5:**
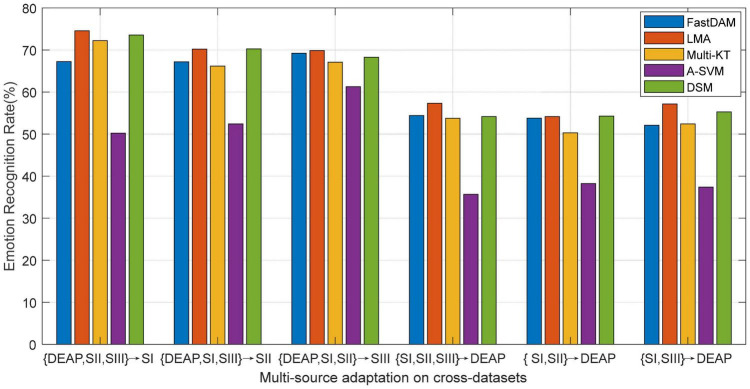
Multi-source adaptation emotion recognition accuracy (SI: Session I, SII: Session II, SIII: Session III).

As reported in preceding works about DA learning, multiple source domains can improve the adaptation performance to some extent by integrating multiple prior knowledge. Nevertheless, in concrete applications, multi-source adaptation also incurs another challenge, i.e., source scalability issue, since multi-source learning could lead to the so-called “negative transfer” problem. In this scenario, how to discriminately exploit multiple sources becomes a challenge worthy to be addressed in the multi-source adaptation learning frameworks. To this end, we will explore in this part the different reliabilities of the prior sources in the emotion recognition task (Tao et al., 2019; Tao and Dan, 2021). We evaluate the performance of all baseline DA methods with multiple prior sources on the designed cross-dataset settings. The average accuracies of all methods are plotted in Figure 5, where A-SVM employs the average prior model.

When there exists very large distribution discrepancy between different domain datasets, it is hard for A-SVM to eliminate the inter-domain distribution bias. Therefore, the results in Figure 5 show that A-SVM is inferior to other multi-source adaptation methods in most settings. A-SVM even has a downgraded performance tendency with the increase of source domains in some scenarios, which indicates the existence of a “negative transfer” phenomenon in A-SVM. Another interesting observation from Figure 5 is that all DA methods except A-SVM achieve more improvement by leveraging multiple source knowledge than by bridging only one source (i.e., cross-subject settings) when the number of source domains increase. This proves that it is beneficial to leverage multiple sources for boosting the recognition performance. Moreover, LMA and DSM conquer others by touching on the top performance, due to their designed weights for discriminately screening the optimal sources. Our method, LMA, obtains more gains over DSM in some scenarios. A possible explanation is that the shared discriminative information among source models in LMA is advantageous to multi-source adaptation learning by utilizing the optimal weight vector.

#### Deep Feature Adaptation

In this subsection, we will particularly evaluate our method, LMA, with deeply extracted features by comparing it with several recently proposed deep adaptation models on cross-dataset emotion recognition using the multi-source settings.

In practical tasks, our method, LMA, can be trained on the deeply transformed features of all domains, which follows the same setup with that in Zhou et al. (2018) and Zhu et al. (2017). Concretely, some pretrained deep model (e.g., VGG16) is first fine-tuned using the source domain, then the deep features can be extracted from EEG signals in both source and target domains with this CNN model, and finally the recognition model would be trained on these extracted features. In the context of our experiments, we denote our methods with the VGG16 model as LMA+VGG16. As for DAN, SDDA, MultiDIAL, and ReverseGrad, we use their released source codes to fine-tune the pre-trained models by respectively using the pre-tuned parameters in their works (Ganin and Lempitsky, 2015; Long et al., 2015). Note that these deep adaptation methods typically aim to learn domain-invariant representations. Different from the deep adaptation frameworks, our proposed method explores to learn a domain-invariant recognition model with strong generalization ability from the source domain to the target domain. Consequently, we expect that our method can further upgrade the recognition performance with the co-learning strategy on the deeply extracted features from some deep model.

**FIGURE 6:**
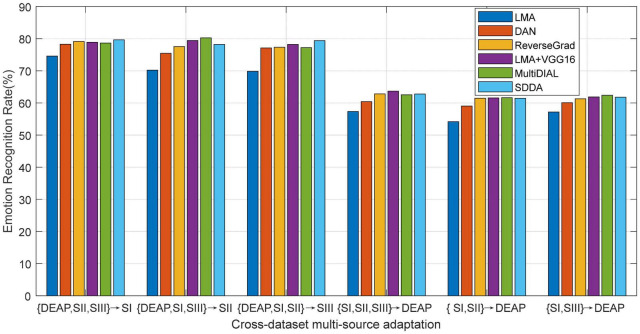
Emotion recognition accuracies of different methods using deeply extracted features (SI: Session I, SII: Session II, SIII: Session III).

We plot the mean results of all methods in Figure 6, from which we can observe that our deep adaptation method LMA+VGG16 significantly outperforms LMA. This indicates the advantage of deep features, which can be attributed to its robust feature representation. Furthermore, LMA+VGG16 also obtains comparable recognition performance with respect to other deep adaptation methods. This may be attributed to the classification-level constraint in LMA, where most of the source discriminative structures are expected to be preserved by the guidance of target classification. In some cases, as shown in Figure 6, LMA+VGG16 even achieves the top performance compared with other deep adaptation frameworks. This phenomenon shows that the proposed LMA can become an effective surrogate to the deep adaptation model by just exploiting the deep features extracted from any one of the state-of-the-art deep models.

#### Parameter Impact

In our method, LMA, there exist three hyper-parameters (i.e., λ, β, and α) that needed to be tuned. These hyper-parameters are mainly used to trade off different components of the LMA framework. We therefore respectively set these parameters into their extreme values to explore the importance of each component in LMA. To this end, we set β = 0 to denote LMA without target feature selection by LMA_NF, set α = η_*a*_ = 0 to denote by LMA_NL the case where LMA ignores latent space representations, and set λ = 0 to denote by LMA_NS the scenario where LMA fails to consider the shared discriminative structures among multiple sources. We evaluate these derived methods on cross-dataset recognition tasks.

The results in Table 2 clearly show that none of the three derived methods can achieve the best performance as that obtained by LMA. This further verifies the valuable contribution of each component to LMA. Specifically, LMA_NL has a significant downgraded manifestation compared with LMA, which, from the opposite side, proves that the utilization of shared latent spaces is very preferable to boosting the performance of LMA; the performance of LMA_NTF is slightly weaker than LMA, i.e., the performance of LMA would be slightly impacted by the target feature selection due to the intrinsic existence of some noise/outlier data in the target data; the inferior performance of LMA_NS proves the importance of the utilization of correlation knowledge among source models in cross-dataset emotion recognition.

**TABLE 2:** Multi-source adaptation emotion recognition accuracies of derived methods as well as LMA.

Method	{DEAP,SII,SIII} →SI	{DEAP,SI,SIII} →SII	{DEAP,SI,SII} →SIII	{SI,SII,SIII} →DEAP	{SI,SII} →DEAP	{SI,SIII} →DEAP
LMA_NF	73.52	69.10	69.58	55.43	52.01	56.22
LMA_NL	69.13	65.36	66.11	52.32	53.16	52.71
LMA_NS	72.68	68.23	68.38	54.69	51.08	54.20
LMA	**74.57**	**70.2**	**69.85**	**57.33**	**54.16**	**57.17**

*Bold denotes the best recognition rates (SI: Session I, SII: Session II, SIII: Session III).*

**FIGURE 7:**
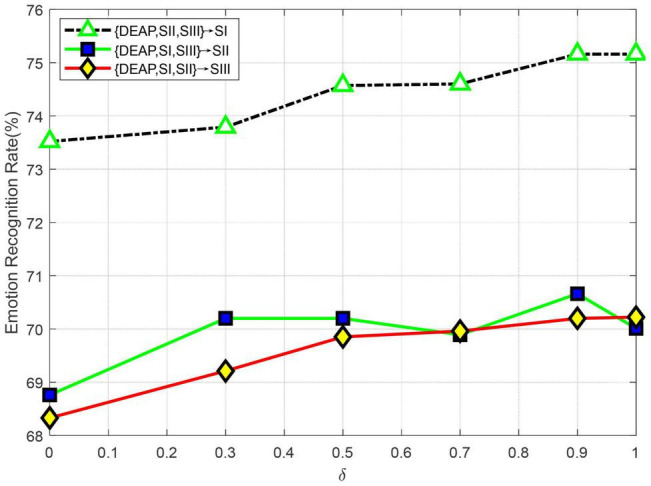
Emotion recognition accuracies with different values of δ (SI: Session I, SII: Session II, SIII: Session III).

Note that in section Emotion Recognition, we use the following combination function to recognize the emotion level of the given test data $x_{i}^{t}$:


$$j=\arg\max_{j}{\left(y_{i}^{t}=\delta f^{s}\left(x_{i}^{t}\right)+\left(1-\delta \right)f^{t}\left(x_{i}^{t}\right)\right)}_{j},$$

where δ ∈ [0,1] is a trade-off parameter, which is empirically set as 0.5 in the preceding trials. In this part, we will further evaluate the impact on LMA with different values of δ in multi-source adaptation scenarios. We plot the recognition accuracy w.r.t. different values of δ in Figure 7. From the curves shown in Figure 7, we can observe the following several interesting results:

(1) Theoretically, δ controls the weight of source classifiers and larger values of δ will make the source classifiers more important in LMA. An extreme case is δ→ 1, where only source classifiers are guaranteed, but the target discriminative information for the test samples is discarded. In this case, all experimental results demonstrate a trend of slight downgrade. This shows the necessity of composite discrimination information by combining both source and target classifiers.
(2) Another extreme case is δ→ 0. In this case, LMA will recognize the emotion state of certain test data by only using the target discriminator, which cannot leverage the prior source information with discriminating power. From Figure 7, we can see that all curves show an obvious upgrade in performance around δ = 0, which shows the importance of multi-source discriminative models in our framework.
(3) We cannot obtain the best performance when δ values are relatively small or large, which shows the significance of exploiting the discriminative information from both source and target classifiers in our method.
(4) After δ > 0.5, we can see that most curves are relatively stable across δ values, which shows that our method is not significantly sensitive to δ > 0.5. Hence, we can empirically set δ = 0.5 in the experiments.

## Conclusion

To deal with the cross-subject/dataset EEG-based emotion recognition task, we proposed a robust LMA. In multiple domain-invariant latent spaces, LMA aims at transferring multi-source knowledge into target learning mainly by leveraging correlation knowledge among source models, which discriminatively screens unimportant prior evidences in sources. The comprehensive experiments performed on two public datasets verify the effectiveness of LMA in dealing with cross-subject/dataset emotion recognition. In most scenarios, our LMA (or LMA-VGG16) obtains the best results or comparable performance with respect to several representative baselines.

Since the implementation of LMA algorithm needs an iterative optimization procedure, how to improve the efficiency of LMA and seek a more efficient algorithm would be an issue worthy of further study in our future research. The unreliable and misleading pseudo labels strategy may be another potential problem in our LMA. Consequently, our successive work would explore how to seamlessly incorporate target label into the framework of LMA.

## Data Availability Statement

The original contributions presented in the study are included in the article/supplementary material, further inquiries can be directed to the corresponding authors.

## Author Contributions

All authors listed have made a substantial, direct, and intellectual contribution to the work, and approved it for publication.

## Conflict of Interest

The authors declare that the research was conducted in the absence of any commercial or financial relationships that could be construed as a potential conflict of interest.

## Publisher’s Note

All claims expressed in this article are solely those of the authors and do not necessarily represent those of their affiliated organizations, or those of the publisher, the editors and the reviewers. Any product that may be evaluated in this article, or claim that may be made by its manufacturer, is not guaranteed or endorsed by the publisher.

## Footnotes

1 While we do not need to limit the number of instances in each source domain, which is identical with that assumed when shaped into the training matrix, for the sake of simplicity, we can extract the same number of training instances from each source domain.

2 It is important to note that the feature mapping function ϕ with respect to each source domain can be completely different from each other.

3 For each source domain, we train one SVM by using the corresponding labeled samples. Then, for each test instance *x*, the decision values from *p* SVM classifiers are converted into the probability values by using the sigmoid function (i.e., g(*t*) = 1/(1 + exp(−*t*)). Finally, we average the *p* probability values as the final pr.
